# Functional results of cataract surgery in the treatment of phacomorphic glaucoma

**DOI:** 10.22336/rjo.2017.37

**Published:** 2017

**Authors:** Andreea Moraru, Gabriela Pînzaru, Anca Moţoc, Dănuţ Costin

**Affiliations:** * “Grigore T. Popa” University of Medicine and Pharmacy, Iaşi, Romania; ** “Prof. N. Oblu” Emergency Hospital, Iaşi, Romania

**Keywords:** cataract surgery, phacomorphic, glaucoma

## Abstract

**Purpose.** Analysis of functional outcome and complications reported in patients diagnosed with phacomorphic glaucoma, in which phacoemulsification or extracapsular extraction of the lens was performed.

**Methods.** The retrospective study included 38 eyes diagnosed with phacomorphic glaucoma. In 25 cases, group 1, the lens was removed by phacoemulsification and in 13 cases, group 2, by extracapsular extraction. Intraocular pressure, visual acuity, and anterior chamber depth were evaluated preoperative and postoperative. The incidence of intra and postoperative complications was analyzed. The minimum follow-up period was 12 months.

**Results.** The mean IOP decreased from the preoperative value of 38.4 +/ - 11.3 mmHg to 13.5 +/ - 3.4 mmHg in group 1 and 11.5 +/ - 3.2 mmHg in group 2. Persistent corneal edema was observed in 32% patients from group 1 and 23% of the patients from group 2. The inflammatory reaction of anterior chamber prevailed in patients from group 2 (46.1%). ACD modified from the preoperative mean of 1.3 +/- 0.5 mm to 2.3 +/- 0.2 mm in both groups. At the end of follow-up in both groups, the average BCVA was 0.6. 18.42% of the cases required long-term topical hypotensive therapy.

**Conclusions.** Both phacoemulsification and extracapsular extraction were safe and effective procedures in the treatment of phacomorphic glaucoma, ensuring a rapid functional recovery and a satisfactory long-term IOP control. Although the rate of immediate postoperative complications and the final functional outcome were better in patients treated with phacoemulsification, not all the cases could be subjected to this type of surgery.

In 2013, the estimated number of people with eyesight deficiencies worldwide was 285 million people, out of which 39 million were blind. 65% of the patients diagnosed with eyesight deficiencies are over 50 years old and the cataract alone constitutes 51% of the causes that lead to blindness [**1**].

Phacomorphic glaucoma is defined as secondary angle-closure glaucoma due to lens thickness increase, which is obstructing the drainage angle. It is characterized by a sudden rise in the intraocular pressure (IOP), which compromises the function of the optic nerve and may lead to irreversible visual loss, if not treated in time [**2**]. 

The mechanism of phacomorphic glaucoma is usually multifactorial. The rapidity of onset and the anatomical predisposition are among the distinguishing features between primary angle closure and phacomorphic angle closure. The onset in phacomorphic glaucoma is sudden and it is due to marked lens intumescence as a result of cataract formation and development of pupillary block in an eye that is otherwise not anatomically predisposed to closure [**3**].

The purpose of our study consisted in the analysis of the functional outcome and complications reported in cases of phacomorphic glaucoma, which underwent extracapsular extraction (ECCE) or phacoemulsification of the lens.

## Material and methods

The retrospective study included 38 eyes diagnosed with phacomorphic glaucoma by subjective complaints of acute pain and redness associated with objective signs such as presence of corneal edema, shallow anterior chamber, an intumescent cataractous lens, and IOP above 21 mmHg.

The preoperative assessments included slit-lamp examination, applanation tonometry, bilateral gonioscopy (when possible) in order to exclude PACG and B-scan ultrasonography to exclude posterior segment pathology. Intraocular pressure, best-corrected visual acuity at distance (BCVA) and anterior chamber depth (ACD) were evaluated preoperative and postoperative. ACD was measured by type A or B immersion ultrasonic biometry.

All the patients were treated with topical beta-blockers, carbonic anhydrase inhibitors, or a combination of the two drugs and oral acetazolamide. In cases in which IOP was higher than 35 mmHg, intravenous mannitol was given. Preoperative intravenous mannitol 20% (1-2 g/ Kg body weight) was administered in all the patients.

The lens extraction was carried out in all the cases. In 25 cases representing group 1, phacoemulsification of the lens nucleus was performed and 13 cases representing group 2 were solved by ECCE. A secondary implantation of an artificial intraocular lens was practiced in 20% (5) of the cases from group 1 and in 30,7% (4) of the cases from group 2. Per primam implantation was performed in all the other cases. Postoperative, patients were treated with topical antibiotic and steroid treatment for 3 to 5 weeks.

The incidence of intraoperative complications and of the complications registered in the immediate postoperative period was analyzed. The minimum follow up period was of 12 months.

## Results 

A total of 38 patients with a mean age of 67,9 years, diagnosed with phacomorphic glaucoma, were included in this study and followed for a period of one year. The preoperative best-corrected visual acuity (BCVA) and the duration of symptoms are summarized in **Table 1** and **Table 2**.

**Table 1:** Preoperative best-corrected visual acuity (BCVA)

BCVA	Number of patients
Light perception	7
Hand motion	16
Counting fingers	12
>0,1	3

**Table 2:** Duration of symptoms

Duration of symptoms (days)	Number of patients (%)
0-10	31 (81,57%)
10-20	5 (13,15%)
>21	2 (5,26%)

Mean preoperative IOP was 38,4 +/ - 11,3 mmHg (range 30 to 60 mmHg).

Gonioscopy showed the presence of angle closure by anterior synechiae in 26,31% of the cases (10 patients).

The extraction of the lens was carried out after lowering the IOP under 21 mmHg, between 2 and 5 days after presentation. 

In 25 cases representing group 1, phacoemulsification of the lens nucleus was performed by using higher quantities of viscoelastics in a “soft shell” technique, in order to deepen the anterior chamber. The nucleus was fragmented by using “stop and chop” technique and a single- piece hydrophobic foldable IOL was injected into the capsular bag or ciliary sulcus in 80% of the cases.

In 13 cases representing group 2, phacoemulsification could not be performed. In these cases, extracapsular cataract extraction was successfully carried out. IOL was injected in sulcus in 70% of the cases. In 20% of the cases (5 eyes) of group 1 and in 30% of the cases (4 eyes) of group 2, secondary implantation of an IOL was performed.

The most important intraoperative challenges were: miosis - 11 eyes (28,94%), fluctuation of the anterior chamber depth - 20 eyes (52,63%), posterior capsular rupture - 3 eyes (7,89%), zonular dehiscence – 4 eyes (10,52%), anterior synechiae > 180º - 10 eyes (26,31%), posterior positive pressure - 2 eyes (5,26%).

In the first postoperative day, severe to moderate corneal edema was present in 8 (32%) patients from group 1 and 3 (23%) patients from group 2. Also, 6 patients (46%) from group 1 and 2 patients (8%) from group 2 presented with severe iritis and a fibrinous pupillary membrane. Two cases from group 2 had mild hyphema (**Table 3**). The complications were solved by administering topical medication. 

**Table 3:** Early postoperative complications in Group 1 and 2

No.	Early postoperative complications	Group 1 (%of patients)	Group 2 (%of patients)
1	Severe to moderate corneal edema	32% (8 cases)	23% (3 cases)
2	Severe iritis with fibrin membrane	8% (2 cases)	46,1% (6 cases)
3	Hyphema	-	15,3% (2 cases)

Late postoperative complications observed were opacification of the posterior capsule in one eye from group 1 and cystoid macular edema in 3 eyes from group 2. Also, 7 eyes, 2 from group 1 and 5 from group 2 developed glaucomatous optic neuropathy (**Table 4**).

**Table 4:** Late postoperative complications in Group 1 and 2

No.	Late postoperative complications	Group 1 (%of patients)	Group 2 (%of patients)
1	Opacification of the posterior capsule	4% (1 eye)	-
2	Cystoid macular edema	23% (3 eyes)	44% (11 eyes)
3	Glaucomatous optic neuropathy	8% (2 eyes)	38,4% (5 eyes)

**Fig. 1:**
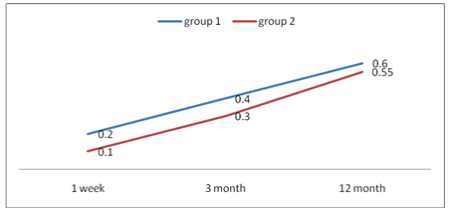
Mean postoperative BCVA values

BCVA status was improved during the first week postoperative. At 3 months after surgery, the mean BCVA in group 1 was 0,4, while that of group 2 was 0,2. At the end of the follow-up in group 1, the average BCVA was 0,6 and 0,55 in group 2. The mean postoperative BCVA values are summarized in **Fig. 1**.

The mean preoperative anterior chamber depth (ACD) was 1,3+/ -0,5 mm. ACD after surgery in both groups had a mean value of 2,3+/ - 0,2 mm, measured by type A or B immersion ultrasonic biometry. The mean postoperative IOP in group 1 was 13,5 +/ - 3,4 mmHg and in group 2, a value of 11,5+/ - 3,2 mmHg was registered. 18,42% of the cases (7 patients) required postoperative topical hypotensive long-term therapy, but none of the cases required glaucoma surgery.

## Discussions

Phacomorphic glaucoma is a type of secondary angle-closure glaucoma, due to a mature and intumescent cataract [**4**]. Historically, this entity has been called phacomorphic glaucoma, but Tham and colab. have pointed out that the use of the word “glaucoma” implies an optic neuropathy and, since the majority of these patients do not have a glaucomatous optic neuropathy, the disease is currently called phacomorphic angle closure [**5**].

The risk factors for phacomorphic angle closure are: age > 60 years, axial length ≤ 23,7 mm, shallow anterior chamber [**6**,**7**].

Control of the high IOP is the first line treatment in a phacomorphic angle closure case, followed by the reduction of corneal edema and cataract surgery. Initial antiglaucomatous medication usually includes beta-blockers, alpha agonists, and carbonic anhydrase inhibitors. Prostaglandin analogues use in such cases has not been reported on a large scale, most likely to avoid their pro-inflammatory effect [**8**]. Many cases require iv mannitol 20% and oral acetazolamide in order to lower the IOP and safely extract the cataractous lens.

Phacoemulsification was attempted in all the eyes included in this study and it has been successfully completed in 25 eyes – group 1. 20 eyes included in group 1 benefited from per primam implantation of the IOL in the capsular bag or the sulcus. Due to the hard nucleus and intraoperative difficulties, we decided to convert the surgery to extracapsular extraction in 13 eyes, included in group 2. 9 of these eyes benefited from per primam implantation of the IOL. 

Corneal edema and AC inflammation with fibrinous reaction during the first few days after surgery are complications frequently found in the early postoperative period [**9**]. In our study, moderate to severe postoperative corneal edema was observed in 32% of the patients from group 1 and in 23% of the patients form group 2, severe iritis with fibrous membrane in 8% of the patients from group 1 and in 46,6% of the patients from group 2, but were successfully treated with topical treatment. 

Studies showed there was a statistically significant association between the duration of the symptoms and the final visual prognosis. A delay higher than 5-10 days from onset to surgery is an important risk factor for an unsatisfactory final VA [**10**].

In our study, 85,57% of the patients reporting a duration of the symptoms of less than 10 days presented a mean BCVA of 0,3, at one month postoperative. In 13,15% of the patients, who reported a duration of the symptoms of 10-20 days, the mean BCVA was 0,15. The worst functional result was registered in the cases in which the delay between the onset and the treatment was more than 20 days. The mean BCVA in this group was 0,05.

Literature data also reported that a duration of symptoms from the onset of more than 10 days is linked with alterations of C/ D ratio [**5**]. At 3 months postoperative, 7 patients included in our study showed an enlargement of the C/ D ratio. 2 of these patients were diagnosed between 10 to 20 days from the onset and 5 after more than 20 days. All these cases required chronic topical hypotensive treatment. This result confirms that a delay of more than 10 days from the diagnosis is a significant risk factor for the development of glaucomatous optic neuropathy.

The final visual prognosis was influenced by late postoperative complications. In 12 cases, the mean final BCVA was less than 0,05 due to opacification of the posterior capsule requiring YAG capsulotomy (1 eye), glaucomatous optic neuropathy (7 eyes), cystoid macular edema (4 eyes).

Data provides postoperative normal reference range values for IOP in both groups analyzed in our study. At the end of the study, the IOP in more than 80% of the cases was maintained under control without needing long-term therapy. Literature data shows that there is a statistically significant difference between the IOP at the first visit and the IOP at the last visit, without any correlations between the IOP at the first visit and the final VA [**10**]. 

## Conclusions

Lens removal through phacoemulsification or ECCE is a safe and effective procedure in the treatment of phacomorphic glaucoma, ensuring a rapid functional recovery and a satisfactory long-term IOP control. Although the rate of immediate postoperative complications and the final functional outcome were better in patients treated by phacoemulsification, not all the cases could be subjected to this type of surgery due to intraoperative difficulties linked to the angle closure.

The final visual outcome is influenced by the delay between the onset of symptoms and the establishment of the diagnosis and by the presence of late postoperative complications, but over 80% of the patients are expected to have a favorable visual outcome. The development of glaucomatous optic neuropathy depends on how soon the treatment is initialized, after the onset of the phacomorphic angle closure.
